# Supplementary material for: Nonsense-mediated mRNA decay factor UPF1 promotes aggresome formation
Source: Nat Commun. 2020 Jun 19;11:3106. doi: 10.1038/s41467-020-16939-6 (PMC7305299; doi:10.1038/s41467-020-16939-6)
Supplement: Supplementary file 1 — Supplementary Information [file 41467_2020_16939_MOESM1_ESM.pdf]

Supplementary Information

Nonsense-mediated mRNA decay factor UPF1 promotes aggresome formation

Park et al.

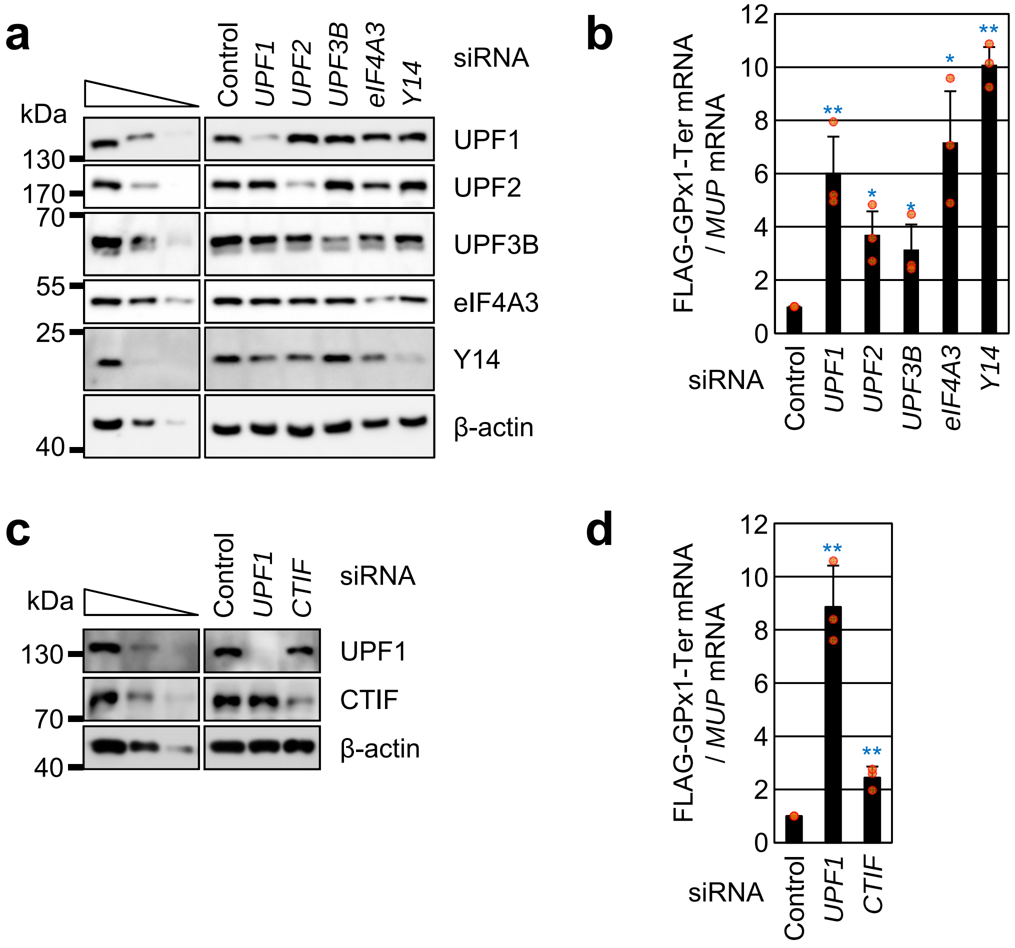

Supplementary Fig. 1 FLAG-GPx1-Ter mRNAs are efficiently degraded by NMD. HeLa cells depleted of the indicated proteins were transfected with an NMD reporter plasmid expressing either FLAG-GPx1-Norm or -Ter and a reference plasmid expressing *MUP* mRNA. **a, c** Western blots illustrating specific downregulation of the endogenous proteins by siRNAs. **b, d** Relative levels of NMD reporter mRNAs determined by qRT-PCRs. Amounts of FLAG-GPx1-Norm or -Ter mRNAs were normalized to the levels of *MUP* mRNAs. The relative ratio of the normalized FLAG-GPx1-Ter mRNA amount to the normalized FLAG-GPx1-Norm mRNA amount in the undepleted cells was arbitrarily set to 1.0. Data are presented as mean values ± SD. Two-tailed, equal-sample variance Student's *t* test was carried out to calculate the *P* values. **P* =0.0119 (*UPF2* siRNA), 0.0313 (*UPF3B* siRNA) and 0.0103 (*eIF4A3* siRNA); ***P* =0.0063 (*UPF1* siRNA in panel **b**), 0.0009 (*UPF1* siRNA in panel **d**), and 0.0039 (*CTIF* siRNA); ***P* <0.0000 (*Y14* siRNA). The exact *P* values are provided in a Source Data File; n = 3; Source data are provided as a Source Data File.

Figure S2

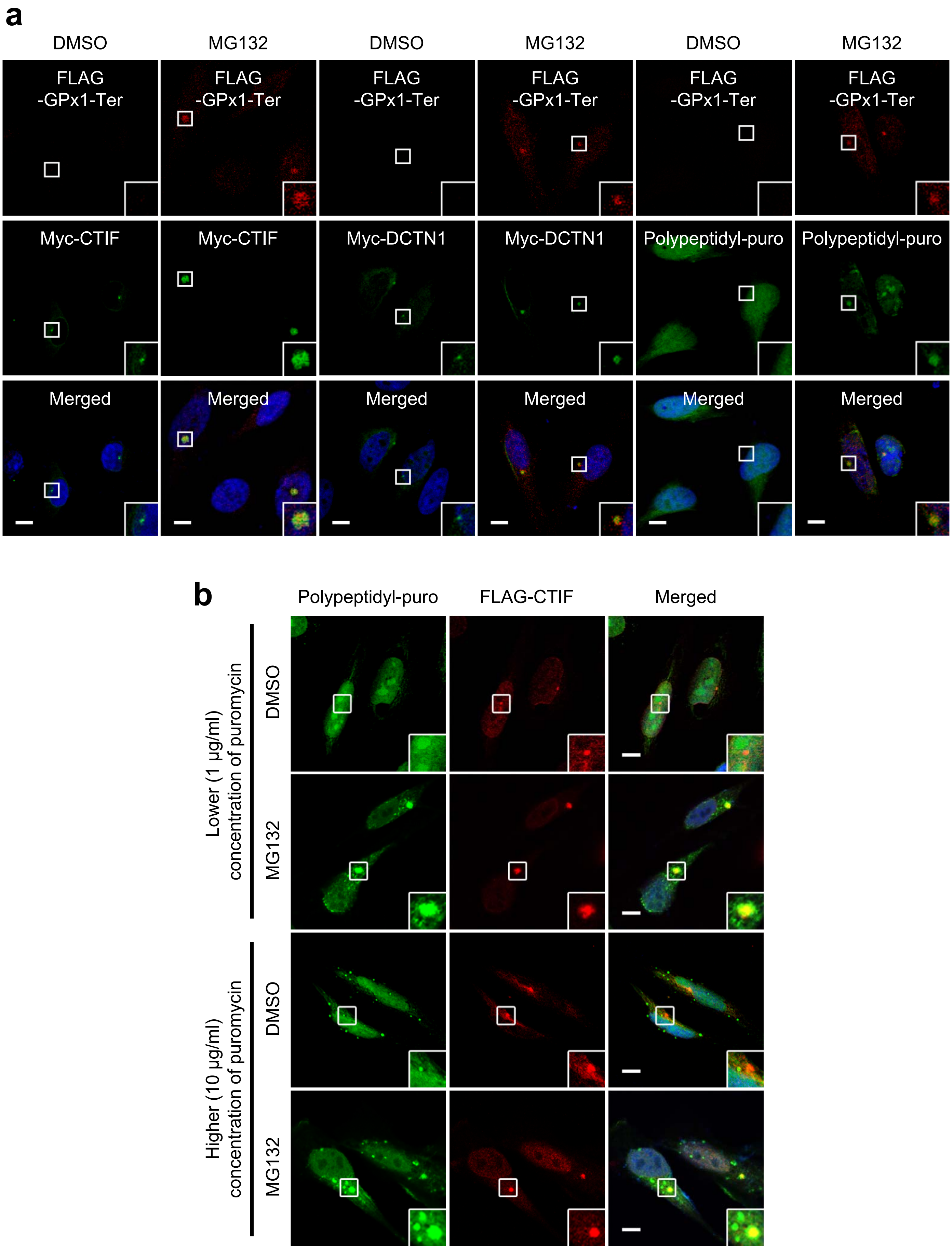
a
DMSO
MG132
DMSO
MG132
DMSO
MG132
FLAG -GPx1-Ter
FLAG -GPx1-Ter
FLAG -GPx1-Ter
FLAG -GPx1-Ter
FLAG -GPx1-Ter
FLAG -GPx1-Ter
Myc-CTIF
Myc-CTIF
Myc-DCTN1
Myc-DCTN1
Polypeptidyl-puro
Polypeptidyl-puro
Merged
Merged
Merged
Merged
Merged
Merged
b
Polypeptidyl-puro
FLAG-CTIF
Merged
Lower (1 µg/ml) concentration of puromycin
DMSO
MG132
Higher (10 µg/ml) concentration of puromycin
DMSO
MG132

Supplementary Fig. 2 FLAG-GPx1-Ter polypeptides colocalize with aggresome-targeting factors or aggresomal proteins. **a** Immunostaining of the FLAG-GPx1-Ter polypeptides (red) and aggresome-targeting factors or aggresomal protein (green) in HeLa cells. The cells were treated with either DMSO or MG132 for 12 h and with puromycin for 1 h before immunostaining. Scale bar, 10 µm; n = 3. **b** Immunostaining of the polypeptidyl-puros (green) and FLAG-CTIF (red) in HeLa cells. As performed in panel **a**, except that the cells were treated with either a lower (1 µg ml^{-1}) or higher concentration (10 µg ml^{-1}) of puromycin for 1 h before immunostaining. Scale bar, 10 µm; n = 3.

Figure S3

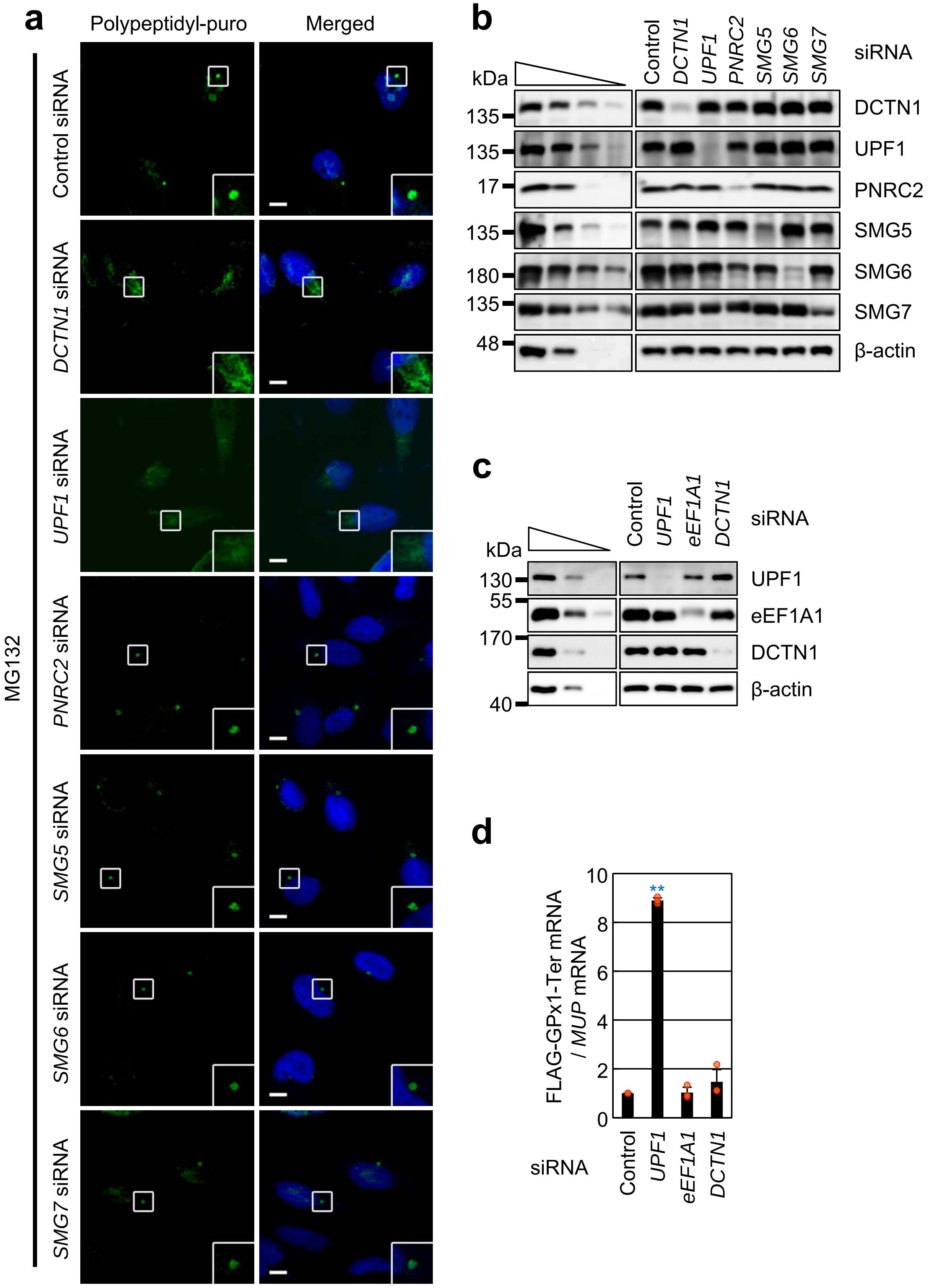

a
Polypeptidyl-puro
Merged
Control siRNA
DCTN1 siRNA
UPF1 siRNA
PNRC2 siRNA
SMG5 siRNA
SMG6 siRNA
SMG7 siRNA
MG132
b
kDa
Control
DCTN1
UPF1
PNRC2
SMG5
SMG6
SMG7
siRNA
135
135
17
135
180
135
48
DCTN1
UPF1
PNRC2
SMG5
SMG6
SMG7
β-actin
c
kDa
Control
UPF1
eEF1A1
DCTN1
siRNA
130
55
170
40
UPF1
eEF1A1
DCTN1
β-actin
d
FLAG-GPx1-Ter mRNA / MUP mRNA
10
8
6
4
2
0
**
siRNA
Control
UPF1
eEF1A1
DCTN1

Supplementary Fig. 3 NMD and the CED-mediated aggresome formation are mechanistically distinct pathways, although they share a common factor, UPF1. **a, b** Effects of downregulation of NMD-related factors on the formation of the aggresome containing polypeptidyl-puro. HeLa cells depleted of the indicated proteins were treated with MG132 for 12 h and puromycin for 1 h before immunostaining. **a** Immunostaining of polypeptidyl-puro. Scale bar, 10 μm; n = 2. **b** Western blotting confirming specific downregulation of the indicated proteins; n = 2. **c, d** The influence of downregulation of CED components on NMD. As performed in Supplementary Fig. 1, except that the cells were depleted of the indicated proteins. **c** Western blotting proving specific downregulation of the indicated proteins; n = 3. **d** Relative levels of FLAG-GPx1-Ter mRNAs determined by qRT-PCR. Data are presented as mean values ± SD. Two-tailed, equal-sample variance Student's *t* test was carried out to calculate the *P* values. $^{**}P < 0.000$. The exact *P* values are provided in a Source Data File; n = 3. Source data are provided as a Source Data File.

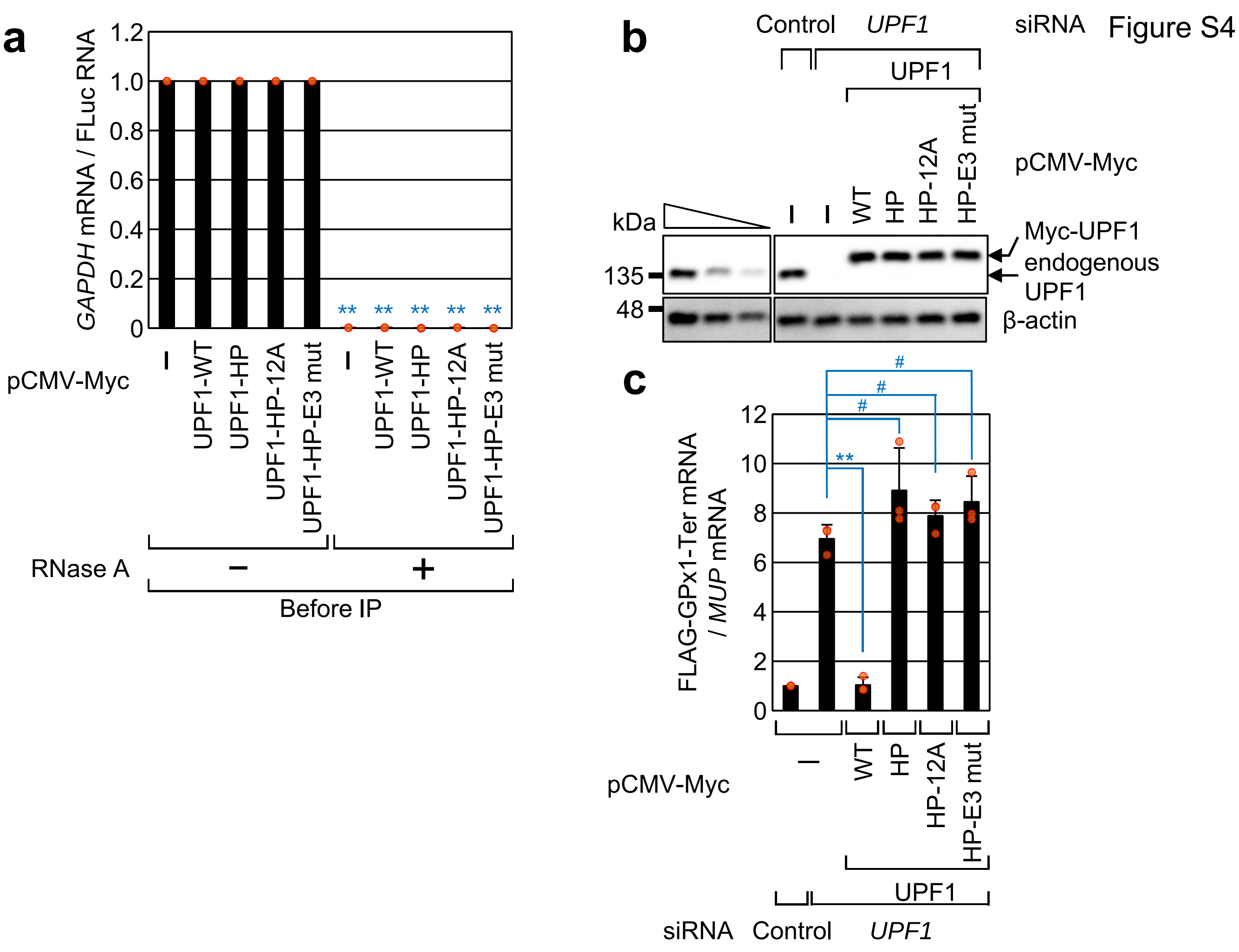

Supplementary Fig. 4 Confirmation of efficient removal of cellular RNAs by treatment with RNase A, and validation of the function of UPF1-WT and its variants in NMD. **a** Efficient removal of cellular RNAs in Fig. 3a. Total-RNA samples were prepared from the extracts either treated or not treated with RNase A before IPs. After that, equal amounts of *in vitro*–synthesized firefly luciferase (FLuc) RNAs were added to the samples as a spike-in to adjust the data for differences among RNA preparations. The amounts of endogenous *GAPDH* mRNA and FLuc RNA were determined by qRT-PCRs. The levels of *GAPDH* mRNA were normalized to those of FLuc RNA. The normalized levels of *GAPDH* mRNA in the extracts not treated with RNase A were arbitrarily set to 1.0. Two-tailed, equal-sample variance Student's *t* test was carried out to calculate the *P* values. $^{**}P < 0.0000$. The exact *P* values are provided in a Source Data File; n = 2. **b, c** HeLa cells stably expressing CFTR-ΔF508 were either depleted or not depleted of endogenous UPF1. One day later, the cells were retransfected with (i) a plasmid expressing NMD reporter mRNA (either FLAG-GPx1-Norm or -Ter), (ii) a plasmid expressing siRNA-resistant UPF1 (either WT or one of the variants), and (iii) a reference plasmid expressing *MUP* mRNA. Two days later, the cells were harvested, and total-RNA samples and total-protein samples were subjected to qRT-PCRs and Western blotting, respectively. **b** Western blotting showing selective downregulation of endogenous UPF1 and comparable expression levels of exogenous UPF1 and endogenous UPF1; n = 3. **c** NMD efficiency of FLAG-GPx1 mRNA. The levels of FLAG-GPx1-Norm or -Ter mRNAs were normalized to the levels of *MUP* mRNAs. The relative ratio of normalized FLAG-GPx1-Ter mRNA to normalized FLAG-GPx1-Norm mRNA in the undepleted cells was arbitrarily set to 1.0. Data are presented as mean values ± SD. Two-tailed, equal-sample variance Student's *t* test was carried out to calculate the *P* values. $^{**}P = 0.0001$. The exact *P* values are provided in a Source Data File; [#]not significant; n = 3; Source data are provided as a Source Data File.

Figure S5

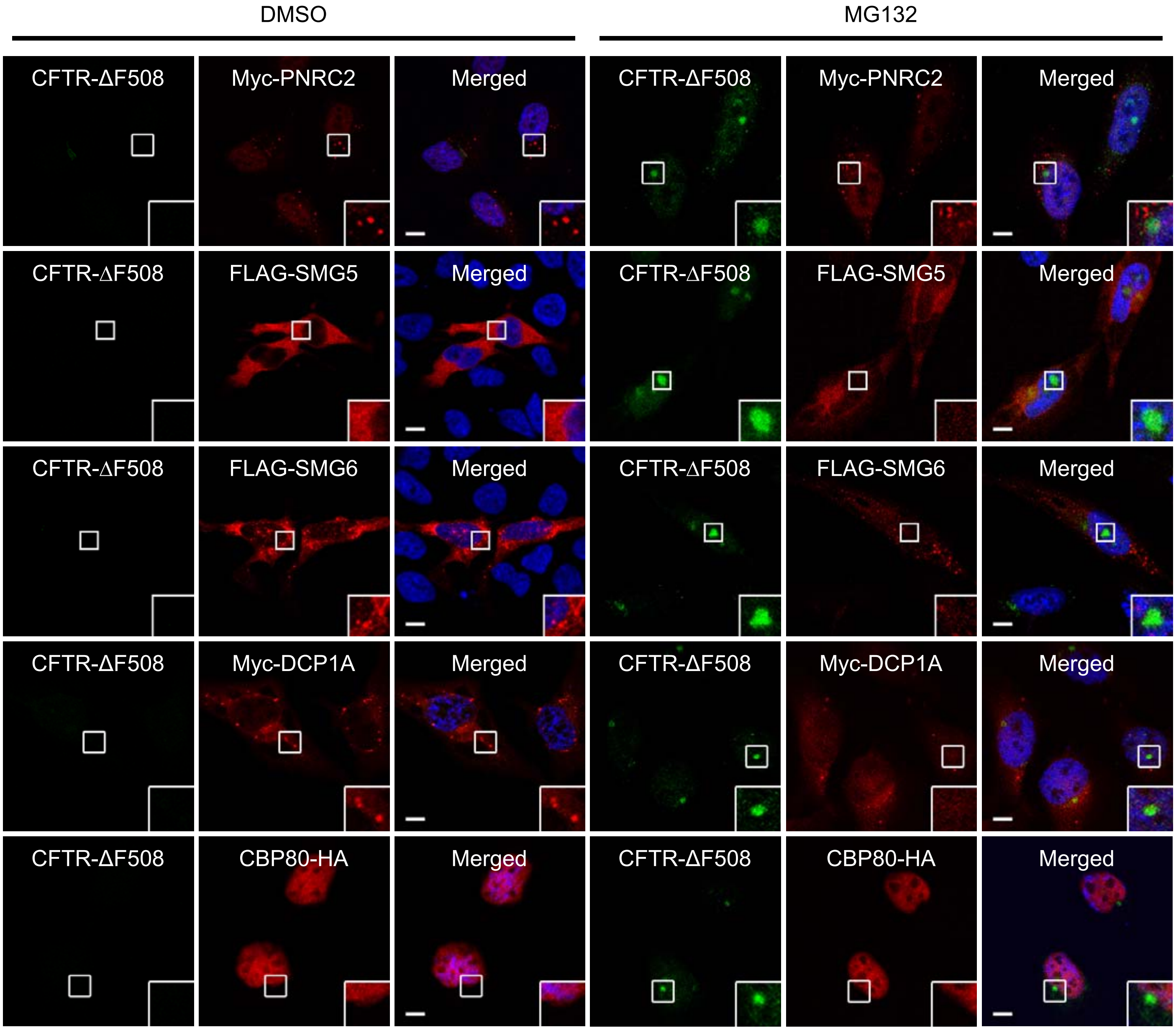

Supplementary Fig. 5 None of the tested NMD-related factors except UPF1 localizes to the aggresome. HeLa cells stably expressing CFTR-ΔF508 were transiently transfected with a plasmid expressing the indicated proteins. The cells were treated with either DMSO or MG132 for 12 h before cell fixation. After that, the cells were subjected to conventional confocal microscopy to analyze the immunostaining of CFTR-ΔF508 (green) and NMD-related factors (red). Scale bar, 10 μm; n = 3.

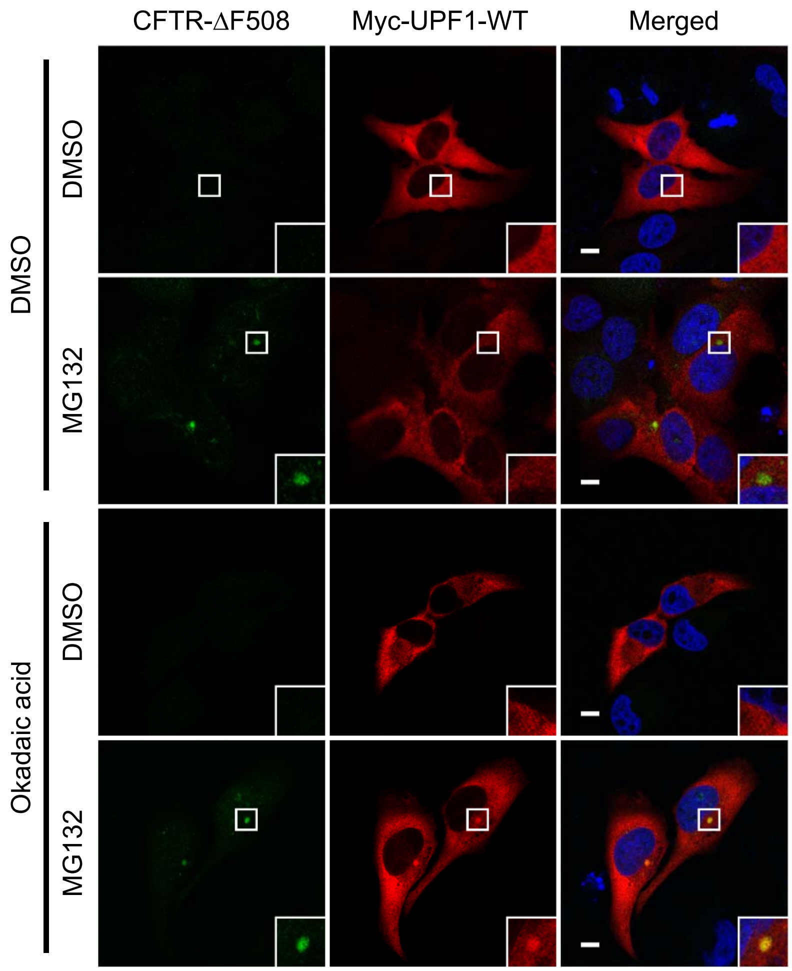

Supplementary Fig. 6 Okadaic acid treatment leads to aggresomal targeting of UPF1-WT. HeLa cells stably expressing CFTR-ΔF508 were transiently transfected with a plasmid expressing Myc-UPF1-WT. The cells were treated with either DMSO or MG132 for 12 h and okadaic acid for 12 h before cell fixation. After that, the cells were subjected to conventional confocal microscopy to analyze the immunostaining of CFTR-ΔF508 (green) and Myc-UPF1-WT (red). Scale bar, 10 μm; n = 2.

Figure S7

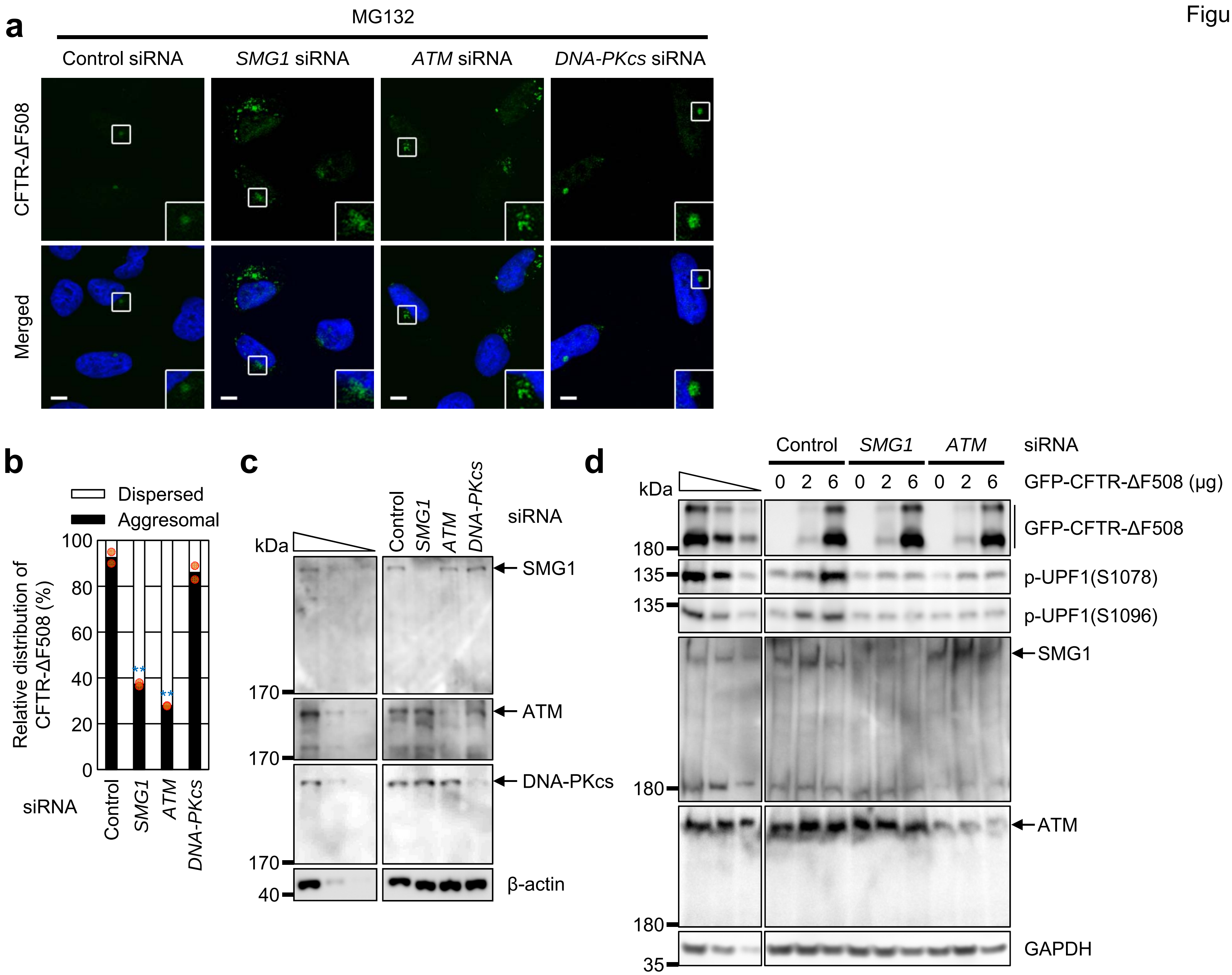

a
MG132
Control siRNA
SMG1 siRNA
ATM siRNA
DNA-PKcs siRNA
CFTR-ΔF508
Merged
b
Dispersed
Aggresomal
Relative distribution of CFTR-ΔF508 (%)
100
80
60
40
20
0
siRNA
Control
SMG1
ATM
DNA-PKcs
c
kDa
Control
SMG1
ATM
DNA-PKcs
siRNA
SMG1
ATM
DNA-PKcs
β-actin
170
170
170
40
d
Control
SMG1
ATM
siRNA
0 2 6 0 2 6 0 2 6
GFP-CFTR-ΔF508 (μg)
kDa
GFP-CFTR-ΔF508
p-UPF1(S1078)
p-UPF1(S1096)
SMG1
ATM
GAPDH
180
135
135
180
180
35

Supplementary Fig. 7 SMG1 and ATM are involved in aggresome formation. HeLa cells stably expressing CFTR-ΔF508 were depleted of the indicated proteins. Then, the cells were treated with MG132 for 12 h before cell fixation. **a** Immunostaining of CFTR-ΔF508. Scale bar, 10 μm; n = 2. **b** The relative percentage of the cells containing either the aggresome or dispersed aggregates of CFTR-ΔF508. As performed in Fig. 2b, except that immunostaining images from panel **a** were analyzed. Two-tailed, equal-sample variance Student's *t* test was carried out to calculate the *P* values. ***P* = 0.0022 (*SMG1* siRNA) and 0.0015 (*ATM* siRNA). More than 100 cells were analyzed from each of two independent experiments. **c** Western blots proving specificity of the downregulation; n = 2. **d** Western blots showing an increase in UPF1 phosphorylation via SMG1 and ATM upon overexpression of a misfolded polypeptide (CFTR-ΔF508); n = 2. Source data are provided as a Source Data File.

Figure S8

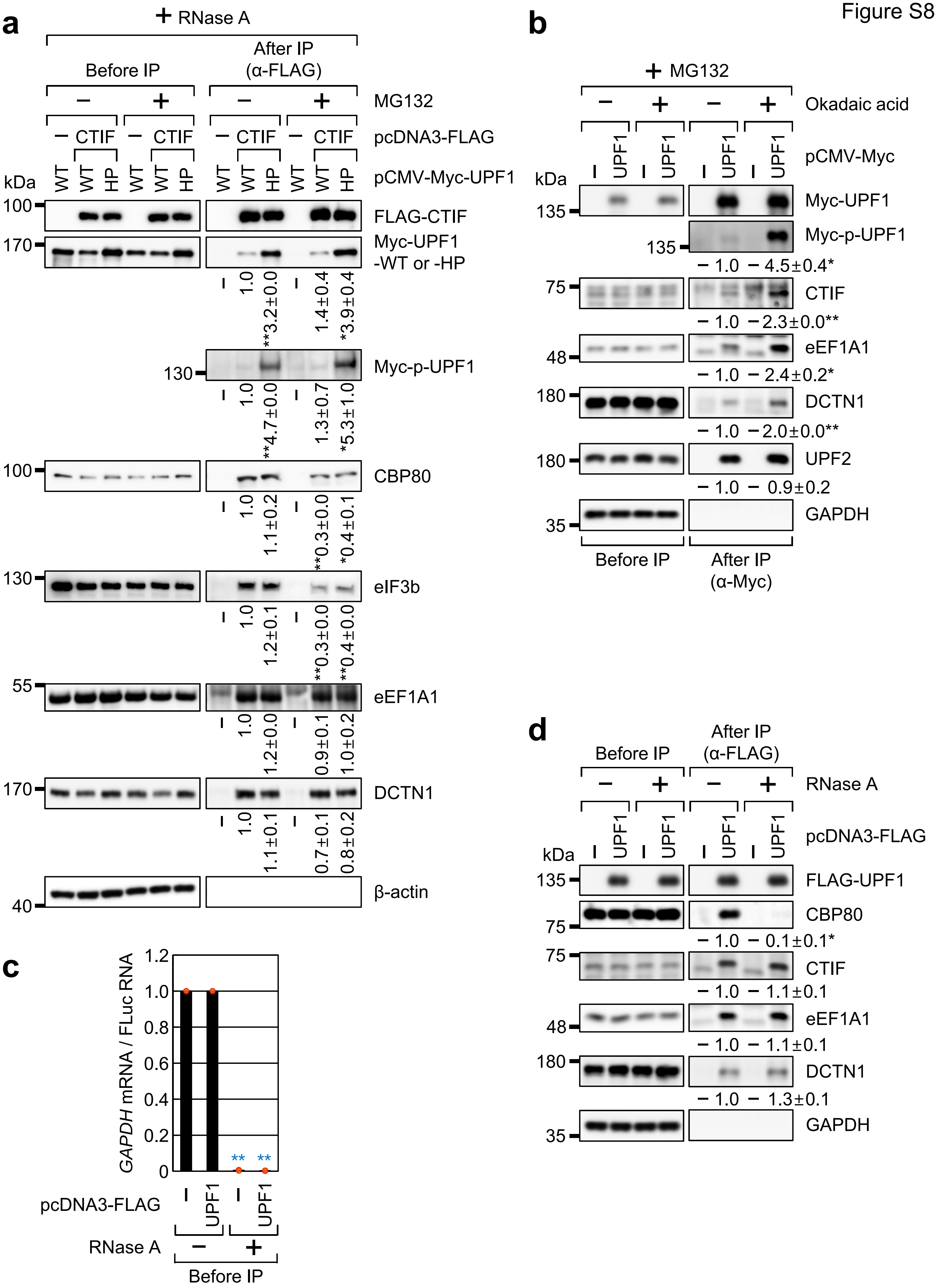
a
+ RNase A
Before IP
After IP (α-FLAG)
MG132
pcDNA3-FLAG
pCMV-Myc-UPF1
kDa
FLAG-CTIF
Myc-UPF1 -WT or -HP
Myc-p-UPF1
CBP80
eIF3b
eEF1A1
DCTN1
β-actin
b
+ MG132
Okadaic acid
pCMV-Myc
Myc-UPF1
Myc-p-UPF1
CTIF
eEF1A1
DCTN1
UPF2
GAPDH
Before IP
After IP (α-Myc)
c
GAPDH mRNA / FLuc RNA
pcDNA3-FLAG
RNase A
Before IP
d
Before IP
After IP (α-FLAG)
RNase A
pcDNA3-FLAG
FLAG-UPF1
CBP80
CTIF
eEF1A1
DCTN1
GAPDH

Supplementary Fig. 8 Hyperphosphorylated UPF1 preferentially binds to the CED complex. **a** IPs of FLAG-CTIF. As performed in Fig. 5a, except that HEK293T cells were transiently cotransfected with plasmids expressing FLAG-CTIF and either Myc-UPF1-WT or –HP. Two-tailed, equal-sample variance Student's *t* test was carried out to calculate the *P* values. **P* < 0.05; ***P* < 0.01. The exact *P* values are provided in a Source Data File; n = 2. **b** IPs of Myc-UPF1 in the lysates of cells either treated or not treated with okadaic acid. HeLa cells stably expressing CFTR-ΔF508 and transiently expressing Myc-UPF1 were either treated or not treated with MG132 for 12 h or okadaic acid for 12 h before cell harvesting. Two-tailed, equal-sample variance Student's *t* test was carried out to calculate the *P* values. **P* =0.0105 (Myc-p-UPF1) and 0.0197 (eEF1A1), ***P* =0.0008 (CTIF) and 0.0007 (DCTN1); n = 2. **c, d** IPs of FLAG-UPF1 in the cell extracts treated or not treated with RNase A. The extract of HEK293T cells transiently expressing FLAG-UPF1 was either treated or not treated with RNase A before IPs with the α-FLAG antibody. **c** qRT-PCRs of endogenous *GAPDH* mRNA showing efficient removal of cellular RNAs. As performed in Supplementary Fig. 4a, and the normalized levels of *GAPDH* mRNA in the extracts not treated with RNase A were arbitrarily set to 1.0. Two-tailed, equal-sample variance Student's *t* test was carried out to calculate the *P* values. ***P* < 0.0000. The exact *P* values are provided in a Source Data File; n = 2. **d** Western blotting of the indicated proteins in the samples before or after IPs of FLAG-UPF1. Two-tailed, equal-sample variance Student's *t* test was carried out to calculate the *P* values. **P* = 0.0129 (CBP80); n = 2; Source data are provided as a Source Data File.

Figure S9

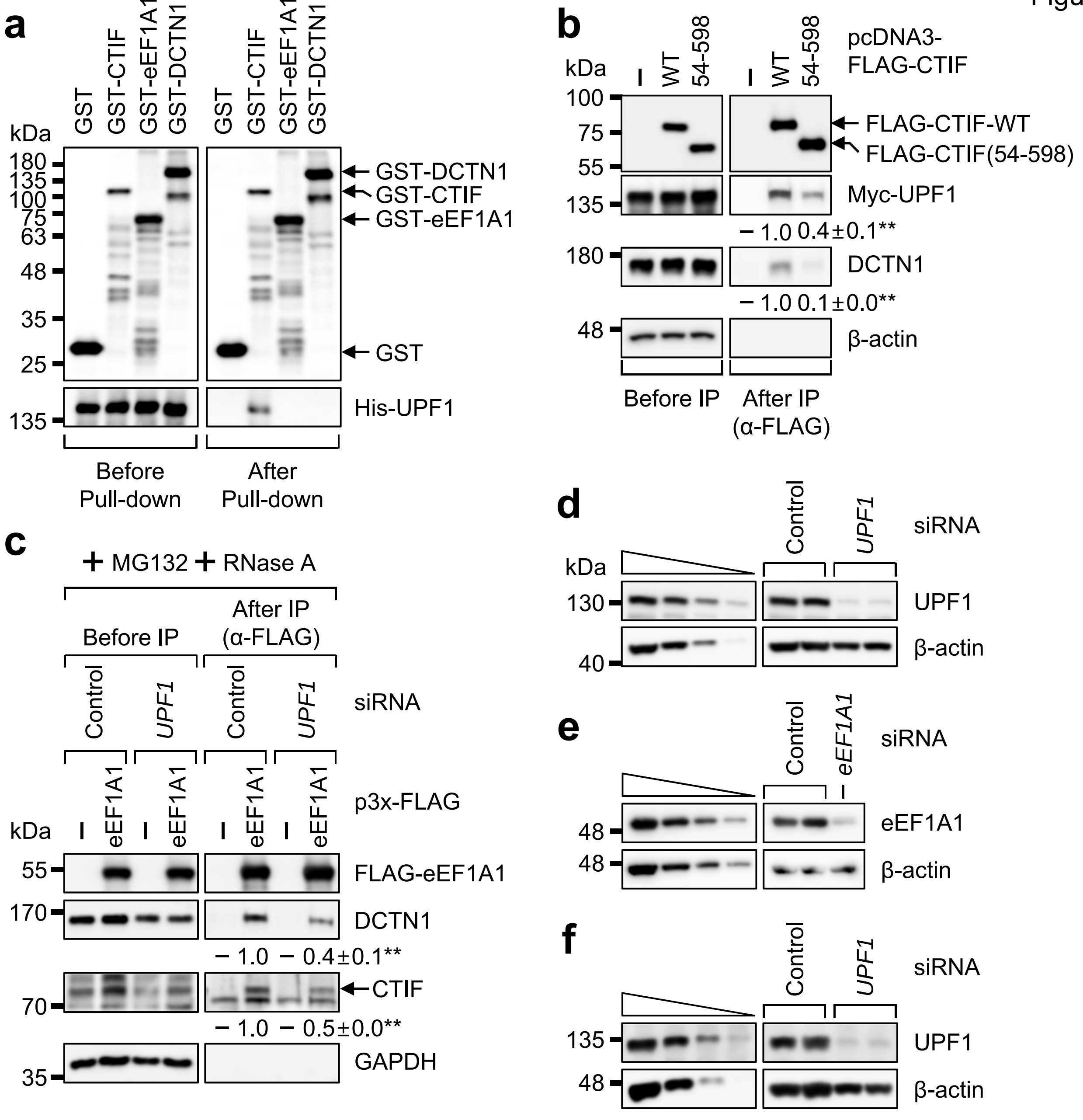

Supplementary Fig. 9 UPF1 promotes the integrity of the CED complex. **a** *In vitro* GST pull-down assays. A lysate of *E. coli* expressing recombinant His-UPF1 was mixed with a lysate of *E. coli* expressing recombinant GST, GST-CTIF, GST-eEF1A1, or GST-DCTN1. After GST pull-down, the resin-bound proteins were subjected to western blotting with an α-GST (upper) or α-His antibody (lower); n = 2. **b** IPs of FLAG-CTIF-WT or FLAG-CTIF(54-598). HEK293T cells were transiently cotransfected with plasmids expressing either FLAG-CTIF-WT or FLAG-CTIF(54-598) and Myc-UPF1. Two-tailed, equal-sample variance Student's *t* test was carried out to calculate the *P* values. $^{**}P$ =0.0077 (Myc-UPF1), $^{**}P < 0.0000$ (DCTN1). The exact P values are provided in Source Data File. n = 2. **c** IPs of FLAG-eEF1A1 in the lysates of cells depleted of UPF1. As performed in Fig. 5c, except that HEK293T cells were transiently transfected with a plasmid expressing FLAG-eEF1A1. Two-tailed, equal-sample variance Student's *t* test was carried out to calculate the *P* values. $^{**}P$ =0.0085 (DCTN1) and 0.0017 (CTIF); n = 2. **d** Western blots proving specific downregulation of UPF1 in Fig. 5c; n = 3. **e** Western blots confirming specific downregulation of eEF1A1 in Fig. 5e; n = 2. **f** Western blots proving specific downregulation of UPF1 in Fig. 5f; n = 2; Source data are provided as a Source Data File.

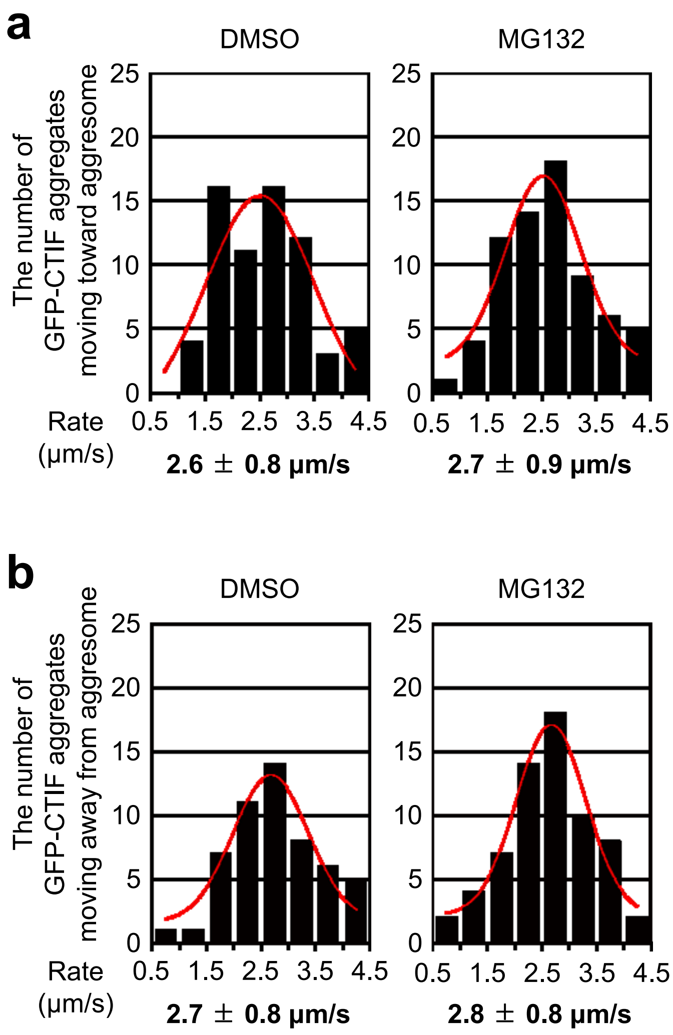

Supplementary Fig. 10 The translocation rate of GFP-CTIF aggregates in the cells either treated or not treated with MG132. **a** The translocation rate of GFP-CTIF aggregates moving toward aggresomes. The cells were visualized within 2 h after the chemical treatment. In total, 69 and 72 independent GFP-CTIF aggregates observed in three individual cells treated with either DMSO or MG132, respectively, were analyzed. The mean values of the translocation rate are depicted below the graph. **b** The translocation rate of GFP-CTIF aggregates moving away from aggresomes. In total, 65 and 53 independent GFP-CTIF aggregates observed in more than three individual cells treated with either DMSO or MG132, respectively, were analyzed.

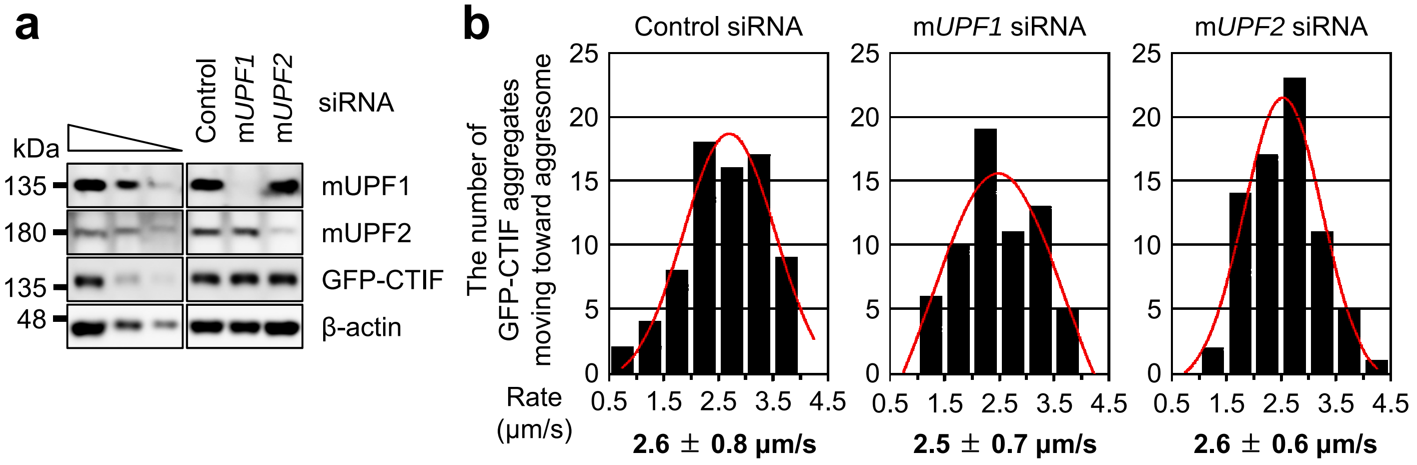

Supplementary Fig. 11 The translocation rate of GFP-CTIF aggregates in the cells either undepleted or depleted of mUPF1 or mUPF2. **a, b** Supporting data for Fig. 6b–e. **a** Western blotting confirming specific downregulation of either mUPF1 or mUPF2. Source data are provided as a Source Data File. **b** The translocation rate of GFP-CTIF aggregates moving toward the aggresome in the cells either undepleted (2.6 ± 0.8 μm s^{-1}; mean ± SD) or depleted of mUPF1 (2.5 ± 0.7 μm s^{-1}) or mUPF2 (2.6 ± 0.6 μm s^{-1}). In total, 74 (three undepleted cells), 64 (five mUPF1-depleted cells), and 73 (five mUPF2-depleted cells) independent GFP-CTIF aggregates were analyzed.

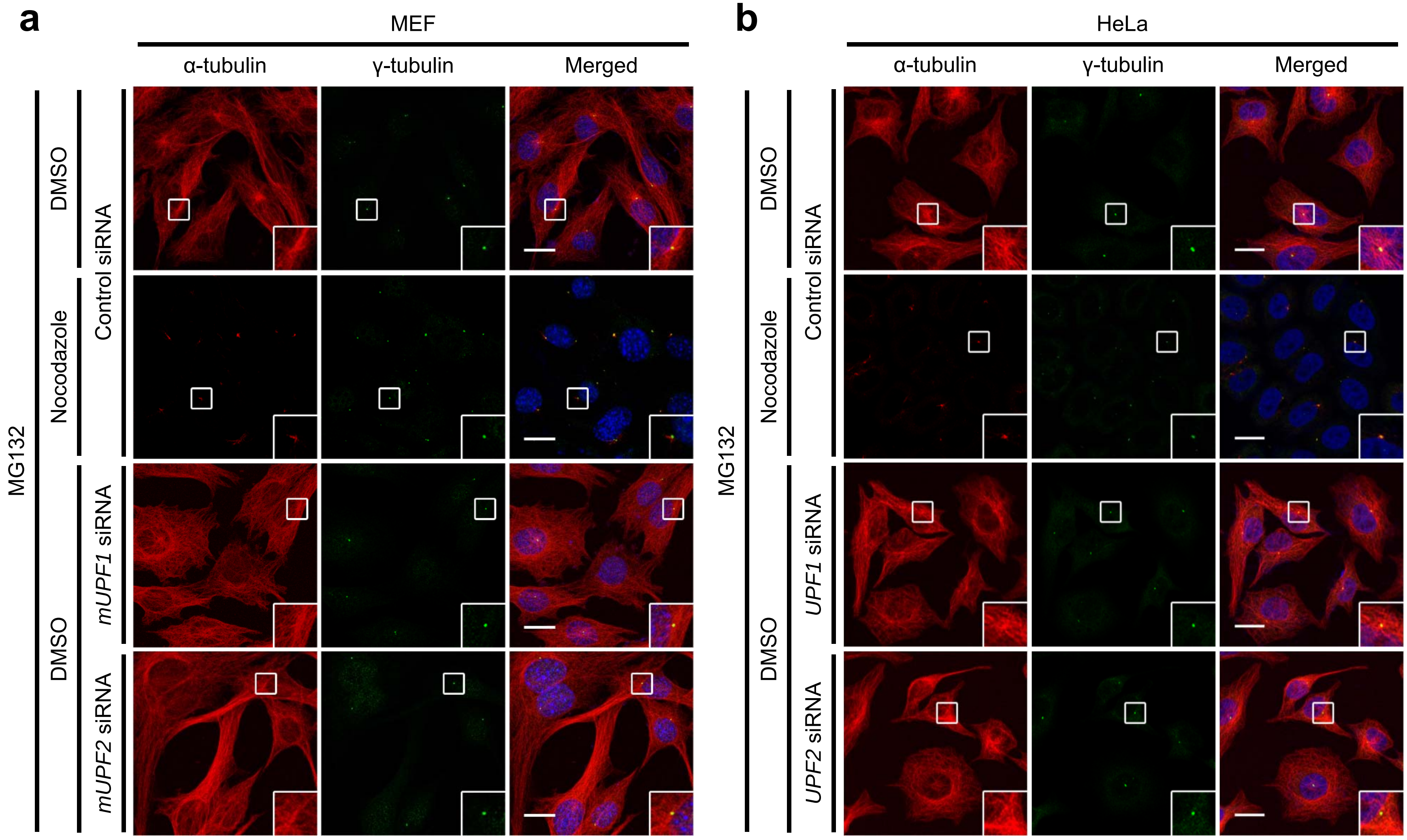

Supplementary Fig. 12 UPF1 downregulation only marginally affected MTOC and microtubule organization. **a** Immunostaining of α-tubulin (a component of microtubules; red) and γ-tubulin (a marker of the MTOC; green) in MEFs depleted of either mUPF1 or mUPF2. As a control, the cells were treated with the microtubule-depolymerizing drug nocodazole. Scale bar, 20 μm; n = 2. **b** Immunostaining of α-tubulin (red) and γ-tubulin (green) in HeLa cells depleted of either UPF1 or UPF2. Scale bar, 20 μm; n = 2.

Figure S13

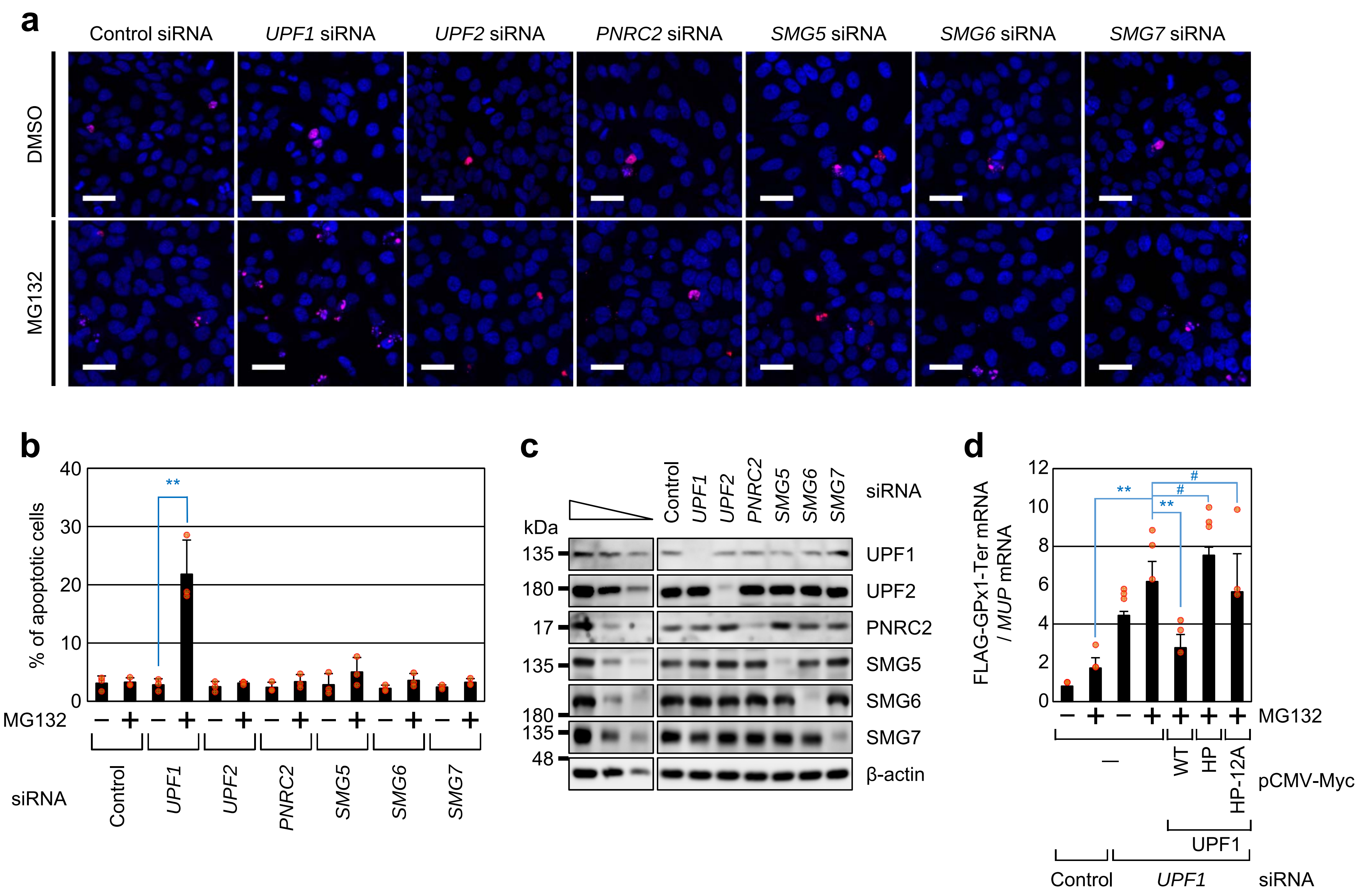

a
Control siRNA
UPF1 siRNA
UPF2 siRNA
PNRC2 siRNA
SMG5 siRNA
SMG6 siRNA
SMG7 siRNA
DMSO
MG132
b
% of apoptotic cells
40
30
20
10
0
**
MG132
siRNA
Control
UPF1
UPF2
PNRC2
SMG5
SMG6
SMG7
c
kDa
Control
UPF1
UPF2
PNRC2
SMG5
SMG6
SMG7
siRNA
135
180
17
135
180
135
48
UPF1
UPF2
PNRC2
SMG5
SMG6
SMG7
β-actin
d
FLAG-GPx1-Ter mRNA / MUP mRNA
12
10
8
6
4
2
0
**
**
#
#
MG132
WT
HP
HP-12A
pCMV-Myc
UPF1
Control
UPF1
siRNA

Supplementary Fig. 13 Inhibition of proteotoxic-stress–induced apoptosis by UPF1 hyperphosphorylation is not relevant to NMD inhibition. **a–c** The influence of downregulation of an NMD factor on the apoptosis induced by proteotoxic stresses. Stably CFTR-ΔF508–expressing HeLa cells either depleted or not depleted of an endogenous NMD factor. The cells were treated with either DMSO or MG132 for 16 h before cell fixation. Scale bar, 50 μm. **a** The population of apoptotic cells was visualized by the TUNEL assay; n = 3. **b** The number of TUNEL-positive cells was determined, and the relative percentage of apoptotic cells is presented. Two-tailed, equal-sample variance Student's *t* test was carried out to calculate the *P* values. $^{**}P = 0.0052$ (*UPF1* siRNA); n = 3. **c** Western blotting showing specific downregulation of an NMD factor; n = 3. **d** NMD efficiency under apoptotic conditions induced by proteotoxic stresses. As performed in Fig. 7a,b, except that the cells were cotransfected with a plasmid expressing either FLAG-GPx1-Norm or -Ter mRNA and a reference plasmid expressing *MUP* mRNA. FLAG-GPx1-Norm and -Ter mRNA levels were normalized to the levels of *MUP* mRNAs. The relative ratio of the normalized FLAG-GPx1-Ter mRNA level to the normalized FLAG-GPx1-Norm mRNA level in the cells not depleted of UPF1 and not treated with MG132 was arbitrarily set to 1.0. Data are presented as mean values ± SD. Two-tailed, equal-sample variance Student's *t* test was carried out to calculate the *P* value. $^{**}P < 0.0089$. The exact P values are provided in Source Data File; $^{\#}$not significant; n = 3; Source data are provided as a Source Data File.
