# Supplementary material for: Nonsense-mediated mRNA decay factor UPF1 promotes aggresome formation
Source: Nat Commun. 2020 Jun 19;11:3106. doi: 10.1038/s41467-020-16939-6 (PMC7305299; doi:10.1038/s41467-020-16939-6)
Supplement: Supplementary file 3 — Reporting Summary [file 41467_2020_16939_MOESM3_ESM.pdf]

nature research

Corresponding author(s): Kim, Yoon Ki

Last updated by author(s): May 22, 2020

Reporting Summary

Nature Research wishes to improve the reproducibility of the work that we publish. This form provides structure for consistency and transparency in reporting. For further information on Nature Research policies, see Authors & Referees and the Editorial Policy Checklist.

Statistics

For all statistical analyses, confirm that the following items are present in the figure legend, table legend, main text, or Methods section.

n/a	Confirmed	
☐	☒	The exact sample size (*n*) for each experimental group/condition, given as a discrete number and unit of measurement
☐	☒	A statement on whether measurements were taken from distinct samples or whether the same sample was measured repeatedly
☐	☒	The statistical test(s) used AND whether they are one- or two-sided<br>*Only common tests should be described solely by name; describe more complex techniques in the Methods section.*
☒	☐	A description of all covariates tested
☒	☐	A description of any assumptions or corrections, such as tests of normality and adjustment for multiple comparisons
☐	☒	A full description of the statistical parameters including central tendency (e.g. means) or other basic estimates (e.g. regression coefficient) AND variation (e.g. standard deviation) or associated estimates of uncertainty (e.g. confidence intervals)
☐	☒	For null hypothesis testing, the test statistic (e.g. F, t, r) with confidence intervals, effect sizes, degrees of freedom and P value noted<br>*Give P values as exact values whenever suitable.*
☒	☐	For Bayesian analysis, information on the choice of priors and Markov chain Monte Carlo settings
☒	☐	For hierarchical and complex designs, identification of the appropriate level for tests and full reporting of outcomes
☐	☒	Estimates of effect sizes (e.g. Cohen's *d*, Pearson's *r*), indicating how they were calculated

Our web collection on statistics for biologists contains articles on many of the points above.

Software and code

Policy information about availability of computer code

Data collection	Light Cycler 480 II machine (Roche) was used for quantitative real-time RT-PCR. Amersham Imager 600 was used for collection of western blotting data. Immunostained cell images were obtained through Zeiss confocal microscope (LSM 510 Meta, LSM 700, and LSM 800). Single-particle tracking in living cells was acquired through multicolor video rate line-scan confocal microscopy. SOLIS imaging software v.4.23.30003.0 (Andor) and LabView scripts were used for recording of living cells and synchronization of recording and scanning, respectively.
Data analysis	Microsoft Excel (Microsoft Office Professional Plus 2016, Microsoft) was used for analyzing of quantitative real-time RT-PCR data. Immunostained cell images were analyzed using Zeiss LSM Image Browser Version 3,5,0,376 and Zen 2.1 (black; Carl Zeiss). For quantitation of cells containing aggresome or dispersed aggregates, cell images were analyzed with Zeiss LSM Image Browser Version 3,5,0,376 and the number of cells containing aggresome or dispersed aggregates was calculated using Microsoft Excel (Microsoft Office Professional Plus 2016, Microsoft). Adobe Photoshop (CS6 version 13.0, Adobe) was used for organizing western blot images and immunostained cell images. Active movement of CTIF aggregates was determined using DiaTrack 3.03 (http://www.diatrack.org) and analyzed with OriginPro 8 (OriginLab) and MATLAB script (2017a, Mathworks). ImageJ v1.51j (National Institutes of Health, Bethesda, MD) was used for subtracting background noise and filtering the data. For the histogram, OriginPro 8 was used for automatically splitting of the data range into equal size of bins. The signal intensities of western blot images were quantitated with the ImageJ v1.42q (National Institutes of Health, Bethesda, MD).

For manuscripts utilizing custom algorithms or software that are central to the research but not yet described in published literature, software must be made available to editors/reviewers. We strongly encourage code deposition in a community repository (e.g. GitHub). See the Nature Research guidelines for submitting code & software for further information.

Data

Policy information about availability of data

All manuscripts must include a data availability statement. This statement should provide the following information, where applicable:

- Accession codes, unique identifiers, or web links for publicly available datasets
- A list of figures that have associated raw data
- A description of any restrictions on data availability

The data supports the findings of this study are available within the article and its Supplementary information files or from the corresponding author upon reasonable request. The source data for Figs. 2b–c, 3a, 4a, 4c, 5a-f, 6e, 7b, and Supplementary Figs. 1a–d, 3b-d, 4a-c, 7b-d, 8a-d, 9a-f, 11a, and 13b-d are provided as a Source Data file.

Field-specific reporting

Please select the one below that is the best fit for your research. If you are not sure, read the appropriate sections before making your selection.

☒ Life sciences ☐ Behavioural & social sciences ☐ Ecological, evolutionary & environmental sciences

For a reference copy of the document with all sections, see nature.com/documents/nr-reporting-summary-flat.pdf

Life sciences study design

All studies must disclose on these points even when the disclosure is negative.

Sample size	Completely independent biological replicates were performed at least two times for all experiments. For quantitation of cell counting, more than 100 cells were analyzed in each experiment, except Fig. 4c. For Fig. 4c, more than 50 cells were analyzed in each experiment. At least two independent biological replicates were analyzed, unless indicated otherwise in the figure legends.
Data exclusions	No data were excluded from analysis
Replication	All experiments were performed at least two independently performed biological replicates.
Randomization	Allocation was random in this study.
Blinding	Blinding was not used except for quantitation and analysis of cells containing aggresome using confocal microscopy. For quantitation of cells containing aggresome or dispersed aggregates, cells were counted and rated in a blinded way by two experienced independent investigators.

Reporting for specific materials, systems and methods

We require information from authors about some types of materials, experimental systems and methods used in many studies. Here, indicate whether each material, system or method listed is relevant to your study. If you are not sure if a list item applies to your research, read the appropriate section before selecting a response.

Materials & experimental systems

n/a	Involved in the study	
☐	☒	Antibodies
☐	☒	Eukaryotic cell lines
☒	☐	Palaeontology
☒	☐	Animals and other organisms
☒	☐	Human research participants
☒	☐	Clinical data

Methods

n/a	Involved in the study	
☒	☐	ChIP-seq
☒	☐	Flow cytometry
☒	☐	MRI-based neuroimaging

Antibodies

Antibodies used	Primary antibodies against the following proteins were commercially available: FLAG (DYKDDDDK; #14793, Cell Signaling Technology, or A8592, Sigma), HA (#3724, Cell Signaling Technology), Myc (9E10; OP10L, Calbiochem or #2272, Cell Signaling Technology), GFP (sc-9996, Santa Cruz Biotechnology), DCTN1 (p150glued; 610474, BD Biosciences), eEF1A1 (CBP-KK1; EF1α;05-235, Merck Millipore), DCP1A (D5444, Sigma), Y14 (RBM8A; MAB2484, Abnova), SMG1 (A300-394A, Bethyl Laboratories), SMG5 (ab33033, Abcam), SMG7 (A302-170A, Bethyl Laboratories), ATM (A300-299A, Bethyl Laboratories), DNA-PKcs (A300-517A, Bethyl Laboratories), phospho-(S/T)Q ATM/ATR substrate (2851, Cell Signaling Technology), puromycin (12D10; MABE343, Merck Millipore), eIF3b (sc-16377, Santa Cruz Biotechnology), γ-tubulin (sc-17788, Santa Cruz Biotechnology), α-tubulin (sc-53030, Santa Cruz Biotechnology), GST (A190-122A, Bethyl Laboratories), His (27-4710-01, GE Healthcare), β-actin (A5441, Sigma), and GAPDH (LF-PA0212, AbFrontier).

The following secondary antibodies were used in this study: a horseradish peroxidase (HRP)-conjugated goat α-mouse IgG antibody (AP124P, Sigma), HRP-conjugated goat α-rabbit IgG antibody (AP132P, Sigma), and HRP-conjugated rabbit α-goat IgG antibody (A5420, Sigma) for western blotting. Alexa Fluor ® 488-conjugated goat anti-mouse IgG (A11017, Invitrogen) and rhodamine-conjugated goat anti-rabbit IgG (31670, Invitrogen) for immunostaining.

Antibodies against the following proteins used for immunostaining, IPs, and western blotting were described previously:

Anti-CTIF
Anti-CTIF was raised in rabbits and tested by western blotting, IP and immunostaining.
Publications:
Kim et al., Genes Dev, 2009
Joori Park et. al., Nat Commun. 2017

Anti-CBP80
Anti-CBP80 was raised in rabbits using the synthetic peptides CRQHKRRSDDDDRSSDRKD (Peptron, Inc., Seoul, Korea) and tested by western blot analysis.
Publications:
Kim et al., Genes Dev, 2009
Jeong K et al., Nucleic Acids Res. 2019

Anti-eIF4A3
anti-eIF4A3 was raised in rabbits using the synthetic peptides ATSGSARKRLLKEEDC (Peptron, Inc., Seoul, Korea) and tested by western blot analysis.
Publications:
Cho et al., Mol cell, 2012
Incheol Ryu et al., Cell Reports. 2019

Anti-UPF1
Anti-UPF1 was from L.E. Maquat, at the University of Rochester Medical Center and tested by western blot analysis.
Publications:
Cho et al., Mol cell, 2012
Jeong K et al., Nucleic Acids Res. 2019

Anti-UPF2
Anti-UPF2 was raised in rabbits using the synthetic peptides CANTNRERRPRYQHPKGAPN (Peptron, Inc., Seoul, Korea) and tested by western blot analysis.
Publications:
Cho et al., Mol cell, 2012
Joori Park et. al., Nat Commun. 2017

Anti-UPF3B
Antibody against human UPF3B was raised in rabbits using the synthetic peptide CKRDRIRNKDRPA (Peptron, Korea) and tested by western blot analysis.
Publications:
Kim et al., Biochem Biophys Res Commun. 2012
Incheol Ryu et al., Cell Reports. 2019

Anti-phospho-S1078-UPF1
Antibodies against phospho-S1078-UPF1 were raised in rabbits using the synthetic peptides CLSQPELpSQDSYLG (Peptron, Inc., Seoul, Korea) and tested by western blot analysis.
Publications:
Cho et al., Mol cell, 2012
Cho H et al., Biochim Biophys Acta. 2013

Anti-phospho-S1096-UPF1
Antibodies against phospho-S1096-UPF1 were raised in rabbits using the synthetic peptides CIDVALpSQDSTY (Peptron, Inc., Seoul, Korea) and tested by western blot analysis.
Publications:
Cho et al., Mol cell, 2012
Cho H et al., Biochim Biophys Acta. 2013

Anti-SMG6
Anti-SMG6 was from S. Ohno, at the University of Yokohama City, and tested by western blot analysis.
Publications:
Okada-Katsuhata Y et al., Nucleic Acids Res. 2012
Junho Choe et al., NAR. 2014

Anti-PNRC2
Antibody against PNRC2 was raised in rabbits and tested by western blot analysis.
Publications:
Cho et al., Mol cell, 2009
Cho et al., Mol cell, 2012

Validation

All commercially available antibodies were validated by the manufacturers.
Valiadation according to website of manufacturers:

Anti-FLAG (DYKDDDDK; #14793, Cell Signaling Technology)
This antibody has been validated using SimpleChIP® Enzymatic Chromatin IP Kits.
Specificity / Sensitivity
DYKDDDDK Tag (D6W5B) Rabbit mAb detects exogenously expressed DYKDDDDK proteins in cells. The antibody recognizes the DYKDDDDK peptide, which is the same epitope recognized by Sigma's Anti-FLAG® antibodies, fused to either the amino-terminus or carboxy-terminus of the target protein.
Source / Purification
Monoclonal antibody is produced by immunizing animals with a synthetic DYKDDDDK peptide.
Publications:
Qin G et al., Nat Commun, 2020
Jana B et al., Life Sci Alliance, 2020

Anti-HA (#3724, Cell Signaling Technology)
This antibody has been validated using SimpleChIP® Enzymatic Chromatin IP Kits.
Specificity / Sensitivity
HA-Tag (C29F4) Rabbit mAb detects exogenously expressed proteins containing the HA epitope tag.
Source / Purification
Monoclonal antibody is produced by immunizing animals with a synthetic peptide containing the influenza hemagglutinin epitope (YPYDVPDYA).
Publications:
Chung SH et al., Mol Ther Methods Clin Dev, 2020
Li N et al., Nucleic Acids Res, 2020

Anti-Myc (9E10; OP10L, Calbiochem or #2272, Cell Signaling Technology)
Specificity / Sensitivity
Myc-Tag Antibody detects recombinant proteins containing the Myc epitope tag. The antibody recognizes the Myc-tag fused to either the amino or carboxy terminus of targeted proteins in transfected cells.
Source / Purification
Polyclonal antibodies are produced by immunizing animals with a synthetic peptide corresponding to residues 410-419 of human c-Myc (EQKLISEEDL). Antibodies are purified by protein A and peptide affinity chromatography.
Publications:
Ruiz-Velasco A et al., Elife, 2020
Fossati M et al., Neuron, 2019

Anti-GFP (sc-9996, Santa Cruz Biotechnology)
GFP (B-2) is a mouse monoclonal antibody raised against amino acis 1-238 representing full length GFP (green fluorsecent protein) of Aequorea victoria origin. GFP Antibody (B-2) is a high quality monoclonal GFP antibody (also designated Green Fluorescent Protein antibody) suitable for the detection of the GFP protein.
Publications:
Shu, Y.N et al., Cardiovasc. Res, 2017
Posavec Marjanović M et al., Nat Struct Mol Biol. 2017

Anti-DCTN1 (p150glued; 610474, BD Biosciences)
p150 [Glued] was identified as a polypeptide associated with cytoplasmic dynein, the minus-end-directed microtubule-based motor protein. p150 [Glued] is also a member of the oligomeric dynactin complex. Microtubule bindings assays with selected constructs of p150 [Glued] indicate that amino acids 39-150 are required for microtubule association. The monoclonal antibody was purified from tissue culture supernatant or ascites by affinity chromatography. The immunogen is Rat p150 [Glued] aa. 3-202. This antibody is routinely tested by western blot analysis.
Publications:
Mara A. Olenick et al., J Biol Chem. 2016
Amy L. Herberta et al., PNAS. 2017

Anti-eEF1A1 (CBP-KK1; EF1α; 05-235, Merck Millipore)
This antibody recognizes EF-1α (Elongation factor-1 alpha), Mr 53 kDa. Occasionally, a 38 kDa protein is also detected in A431 cell lysate. Detect EF1α using this Anti-EF1α Antibody, clone CBP-KK1 validated for use in IP & WB. Crude calmodulin-binding proteins from Trypanosoma brucei isolated using calmodulin-affinity chromatography.
Publications:
Rebecca Frum et al., J Proteome Res. 2007
Corinne von Kanel et al., eLIFE. 2020

Anti-DCP1A (D5444, Sigma)
Anti-DCP1A (C-terminal) antibody produced in rabbit. Anti-DCP1A antibody produced in rabbit is suitable for immunoprecipitation at a working concentration of 5-10 µg using cell lystes of HEK-293T, indirect immunofluorescence at 2-5 µg/mL using paraformaldehyde-fixed NIH-3T3 cells over-expressing human DCP1A or using paraformaldehyde-fixed HEPG2 cells and western blot analysis at 1-2 µg/mL working concentration using cell lysates of HEK-293T. It was used as a primary antibody at a working dilution of 1:200 in the immunofluorescence experiment of HeLa cells treated with 5-fluorouracil to study the assembly of stress granules based on RNA incorporation. Applications in which this antibody has been used successfully.
Publications:
J. Liu et al., Biochemical and Biophysical Research Communications, 2019
Joori Park et. al., Nat Commun. 2017

Anti-Y14 (RBM8A; MAB2484, Abnova)
Mouse monoclonal antibody raised against RBM8A, clone 4C4. Immunogen is human RBM8A and host is mouse.

Quality Control Testing: Antibody Reactive Against Recombinant Protein.
Specificity: Detects a band of approximately 22 KDa
Publications:
Incheol Ryu et al., Cell Reports. 2019
Jeong K et al., Nucleic Acids Res. 2019

Anti-SMG1 (A300-394A, Bethyl Laboratories)
Rabbit anti-SMG1 is a polyclonal antibody. The antibody was affinity purified using an epitope specific to SMG1 immobilized on solid support. The epitope recognized by A300-394A maps to a region between residues 2925 and 2975 of human PI-3-Kinase-related kinase SMG1 using the numbering given in entry NP_055907.3 (GeneID 23049).
Presumed reactivity: Based on 100% sequence identity, this antibody is predicted to react with Mouse.
Publications:
Cho H et al., Biochim Biophys Acta. 2013
Seyedali A et al., RNA. 2014

Anti-SMG5 (ab33033, Abcam)
Anti-SMG5 antibody is rabbit polyclonal antibody to SMG5. Synthetic peptide conjugated to KLH derived from within residues 1000 to the C-terminus of human SMG5.
Publications:
Park OH et al., Genes Dev. 2016
Tatsuaki Kurosaki et al., NSMB. 2018

Anti-SMG7 (A302-170A, Bethyl Laboratories)
Anti-SMG7 is a Rabbit Polyclonal antibody. Antibody was affinity purified using an epitope specific to SMG7 immobilized on solid support. The epitope recognized by A302-170A maps to a region between residue 1087 and 1137 of human suppressor with morphogenetic effect on genitalia protein 7 (ever shorter telomeres 1C) using the numbering given in entry NP_775179.1 (GeneID 9887).
Publications:
Hongwei Luo et al., Cell Discov. 2016
Jungyun Park et al., Nat Commun. 2019

Anti-ATM (A300-299A, Bethyl Laboratories)
Anti-AMT is a Rabbit Polyclonal antibody. Antibody was affinity purified using an epitope specific to MAD2 immobilized on solid support. The epitope recognized by A300-299A maps to a region between residues 2550 and 2600 of human ataxia telangiectasia mutated using the numbering given in SwissProt entry Q13315 (GeneID 472).
Publications:
Cho H et al., Biochim Biophys Acta. 2013
Moises A. Serrano et al., Oncogene. 2013

Anti-DNA-PKcs (A300-517A, Bethyl Laboratories)
Anti-DNA-PKcs is a Rabbit Polyclonal antibody. Antibody was affinity purified using an epitope specific to DNA-PKcs immobilized on solid support. The epitope recognized by A300-517A maps to a region between residues 2050 and 2100 of human DNA-Dependent Protein Kinase, catalytic subunit using the numbering given in entry NP_008835.5 (GeneID 5591).
Publications:
Zhijie Liu et al., Cell. 2014
Yifan Wang et al., Genes Dev. 2016

Anti-phospho-(S/T)Q ATM/ATR substrate (2851, Cell Signaling Technology)
Specificity / Sensitivity
Phospho-(Ser/Thr) ATM/ATR Substrate Antibody detects endogenous levels of proteins containing the ATM/ATR substrate motif. This antibody preferentially binds peptides and proteins that contain phospho-Ser/Thr preceded by Leu or similar hydrophobic amino acids at the -1 position and followed by Gln at the +1 position. The antibody does not cross-react with corresponding nonphosphorylated sequences or with other phospho-Ser/Thr-containing motifs.
Source / Purification
Polyclonal antibodies are produced by immunizing animals with synthetic phospho-(Ser/Thr) ATM/ATR substrate peptides. Antibodies are purified by protein A and peptide affinity chromatography.
Publications:
Jeong K et al., Nucleic Acids Res. 2019
Ana Rio-Machin et al., Nat Commun. 2020

Anti-puromycin (12D10; MABE343, Merck Millipore)
Anti-Puromycin antibody, clone 12D10, detects puromycin incorporated into protein. Monoclonal antibodies to puromycin may be used with standard immunochemical methods. Demonstrated to react with Human test sample, preincubated with Puromycin. Predicted to react with all species when test sample is incubated with Puromycin. Evaluated by Western Blotting in HEK293 cell lysates treated with Puromycin and Cycloheximide, or with Puromycin only.
Publications:
Yamada SB et al., Nat Neurosci. 2019
Wang et al., PNAS. 2016

Anti-eIF3b (sc-16377, Santa Cruz Biotechnology)
Anti-eIF3b (N-20) is an affinity purified goat polyclonal antibody raised against a peptide mapping at the N-terminus of eIF3b of human origin.
Publications:
Wang, X. et al., Nature. 2014
Marc D. Panas et al., Methods. 2015

Anti-γ-tubulin (sc-17788, Santa Cruz Biotechnology)
γ-Tubulin (D-10) is a mouse monoclonal antibody raised against amino acids 269-451 mapping at the C-terminus of γ Tubulin of human origin.
Publications:
Taiki Tsutsui et al., J Biol Chem. 2013
Fan Lai et al., Nature. 2016

Anti-α-tubulin (sc-53030, Santa Cruz Biotechnology)
α-Tubulin (YOL1/34) is a rat monoclonal antibody raised against full length purified α Tubulin of Saccharomyces cerevisiae origin. α-Tubulin (YOL1/34) is recommended for detection of α Tubulin of mouse, rat, human and yeast origin.
Publications:
Shoemaker, C.J., et al., PLoS Biol. 2019
Li, H., et al., Neuron. 2019

Anti-GST (A190-122A, Bethyl Laboratories)
Anti-GST is a Rabbit Polyclonal antibody. Rabbits were immunized with Glutathione-S-Transferase (GST) from Schistosoma japonicum. Antibody was isolated by affinity chromatography using GST immobilized on solid support.
Publications:
Park OH et al., Mol Cell. 2019
Xingcheng Chen et al., Cell Signal. 2017

Anti-His (27-4710-01, GE Healthcare)
Anti-His Antibody is an IgG2 subclass of monoclonal antibody directed against six histidine residues, which are commonly used as an affinity tag for recombinant fusion proteins. The antibody is unconjugated to offer the flexibility of detection with a secondary antibody conjugated with either horseradish peroxidase or alkaline phosphatase. The antibody provides high sensitivity with low background. Anti-His Antibody is quality tested to detect a GST-(histidine)6-tagged protein at levels of » 50 ng in ELISA and 100 ng in Western blots.
Publications:
Xu X et al., Nat Commun. 2017
Kaufmann T et al., J Cell Sci. 2016

Anti-β-actin (A5441, Sigma)
Monoclonal Anti-β-Actin (mouse IgG1 isotype) is derived from the AC-15 hybridoma produced by the fusion of mouse myeloma cells and splenocytes from an immunized mouse. Actin is one of the most conserved eukaryotic proteins, it is expressed in mammals and birds as at least six isoforms. Four of them represent the differentiation markers of muscle tissues and two are found practically in all cells. There are three α-actins (α-skeletal, α-cardiac, and α-smooth muscle), one β-actin (β-nonmuscle), and two γ-actins (γ-smooth muscle and γ-non-muscle). Actin isoforms show >90% overall sequence homology, but only 50–60% homology in their 18 NH2-terminal residues. The NH2-terminal region of actin appears to be a major antigenic region and may be involved in the interaction of actin with other proteins such as myosin.
Immunogen:
slightly modified β-cytoplasmic actin N-terminal peptide, Ac-Asp-Asp-Asp-Ile-Ala-Ala-Leu-Val-Ile-Asp-Asn-Gly-Ser-Gly-Lys, conjugated to KLH.
Application:
Monoclonal Anti-β-Actin antibody produced in mouse has also been used for immunofluorescence staining, immunoblotting and immunohistochemistry. Anti-actin antibody was used as a loading control for western blot analysis of protein lysates, immunoprecipitated proteins.
Publications:
Ming-Yuh Shiau et al., Sci Rep. 2019.
Kazutoshi Takahashi et al., Cell. 2006

Anti-GAPDH (LF-PA0212, AbFrontier)
Anti-GAPDH is Rabbit polyclonal to GAPDH. Recombinant human protein purified from E.coli and Protein A purified.
Publications:
Kim et al., Genes Dev, 2009
Jeong K et al., Nucleic Acids Res. 2019

Antibodies against the following proteins used for immunostaining, IPs, and western blotting were described previously:
CTIF and CBP80 (Kim et al., Genes Dev, 2009); eIF4A3, UPF1, UPF2, phospho-S1078-UPF1, and phospho-S1096-UPF1 (Cho et al., Mol cell, 2012); UPF3B (Kim et al., BBRC, 2012); SMG6(Okada-Katsuhata et al., NAR, 2012); and PNRC2 (Cho et al., Mol cell, 2009). In this study, rabbit polyclonal anti-CTIF, CBP80, eIF4A3, UPF1, UPF2, UPF3B, PNRC2 ,and SMG6 antibodies were validated by western blotting showing specific downregulation and/or coimmunopurification of each proteins, respectively.

Eukaryotic cell lines

Policy information about cell lines

Cell line source(s)	HeLa and HEK293T cells are purchased from ATCC. MEF (mouse embryonic fibroblast) cells were obtained from Prof. Byung-Yoon Ahn (Korea university, Seoul, Korea), which was originally purchased from ATCC. HeLa cells stably expressing CFTR-ΔF508 was described previously (Park et al., Nat commun, 2017).
Authentication	HeLa , HEK293T, and MEF cells were obtained from a commercial vendor which is verified by manufacturer (ATCC). HeLa cells stably expressing GFP-CFTR-ΔF508 were verified by qRT-PCR and immunostaining using two oligonucleodites [5'-

GGAGTACAACTACAACAGCC-3' (sense) and 5'-CAGCAGGACCATGTGATCGC-3' (antisense)] and anti-GFP (sc-9996, Santa Cruz Biotechnology), respectively.

Mycoplasma contamination	To minimize mycoplasma contamination, we employed Plasmocin (Invivogen). We used MycoAlert PLUS Mycoplasma detection kit (Lonza) to ensure that the cells used in this study had no mycoplasma contamination.
Commonly misidentified lines (See ICLAC register)	No commonly misidentified cell lines were used in the study.
