# Supplementary material for: Nonsense-mediated mRNA decay factor UPF1 promotes aggresome formation
Source: Nat Commun. 2020 Jun 19;11:3106. doi: 10.1038/s41467-020-16939-6 (PMC7305299; doi:10.1038/s41467-020-16939-6)
Supplement: Supplementary file 4 — Description of Additional Supplementary Files [file 41467_2020_16939_MOESM4_ESM.pdf]

Description of Additional Supplementary Files

File Name: Supplementary Data 1
Description: SiRNA sequences used in this study.

File Name: Supplementary Movie 1
Description: Video for Fig. 6a.

File Name: Supplementary Movie 2
Description: Video for Supplementary Fig. 10.

File Name: Supplementary Movie 3
Description: Video for Fig. 6b–e and Supplementary Fig. 11. Individual GFP-CTIF signals in the mUPF1-depleted cells were not as bright as those in the undepleted or mUPF2-depleted cells. To better visualize GFP-CTIF movements, we enhanced the contrast of images of the mUPF1-depleted cell, resulting in a much larger aggresome as depicted in the movie.
